# Color coded metadevices toward programmed terahertz switching

**DOI:** 10.1038/s41377-024-01495-1

**Published:** 2024-06-25

**Authors:** Weibao He, Xiang’ai Cheng, Siyang Hu, Ziheng Ren, Zhongyi Yu, Shun Wan, Yuze Hu, Tian Jiang

**Affiliations:** 1 College of Advanced Interdisciplinary Studies, National University of Defense Technology, Changsha, China; 2 Nanhu Laser Laboratory, National University of Defense Technology, Changsha, China; 3 Hunan Provincial Key Laboratory of High Energy Laser Technology, National University of Defense Technology, Changsha, China; 4 Institute for Quantum Science and Technology, College of Science, National University of Defense Technology, Changsha, China

**Keywords:** Metamaterials, Terahertz optics

## Abstract

Terahertz modulators play a critical role in high-speed wireless communication, non-destructive imaging, and so on, which have attracted a large amount of research interest. Nevertheless, all-optical terahertz modulation, an ultrafast dynamical control approach, remains to be limited in terms of encoding and multifunction. Here we experimentally demonstrated an optical-programmed terahertz switching realized by combining optical metasurfaces with the terahertz metasurface, resulting in 2-bit dual-channel terahertz encoding. The terahertz metasurface, made up of semiconductor islands and artificial microstructures, enables effective all-optical programming by providing multiple frequency channels with ultrafast modulation at the nanosecond level. Meanwhile, optical metasurfaces covered in terahertz metasurface alter the spatial light field distribution to obtain color code. According to the time-domain coupled mode theory analysis, the energy dissipation modes in terahertz metasurface can be independently controlled by color excitation, which explains the principle of 2-bit encoding well. This work establishes a platform for all-optical programmed terahertz metadevices and may further advance the application of composite metasurface in terahertz manipulation.

## Introduction

Miniaturization and higher integration of on-chip optical systems^1^ necessitate more device adaptability, versatility, and data storage capacity^2–5^. Metasurfaces, a form of ultrathin planar optical component, have emerged as an advanced scheme for controlling light and enabling a wide range of optical applications in previous studies^6–12^. Especially for terahertz waves with high-capacity communications, metasurfaces provide a potential solution to the lack of functional devices. Terahertz metasurfaces have addressed a great number of scientific and technological challenges, delivering essential assistance for boosting the development of terahertz functional devices^13–19^. Scalable metasurfaces with individually programmable elements, simultaneous amplitude and phase control, and gigahertz-speed reconfiguration are required to fulfill the full promise of reconfigurable features and enable sophisticated electromagnetic transformations. Reconfigurability in active terahertz metasurfaces has been demonstrated with liquid crystal^20–22^, phase-change materials^23–25^, mechanical systems^26,27^, and two-dimensional materials^28,29^. These approaches, however, usually have a poor response time, are not scalable, and require substantial external stimuli. To increase the capacity of information transmission, ultrafast terahertz switching and programmability will need to be combined for the next generation of integrated devices.

Light-driven reconfigurable metasurfaces that carry modulation information in amplitude, phase, frequency, and polarization are receiving a great deal of attention and research due to their contactless, succinct construction and ultrafast response-ability^30–38^. The ultrafast modulation behavior benefits significantly from the photogenerated carrier relaxation dynamics of active materials, in particular intrinsic epitaxial silicon (Si) and amorphous germanium, which have lower preparation costs with relaxation times in the order of nanoseconds and picoseconds^39–42^. These materials are advantageous in the preparation of ultrafast terahertz metadevices. In general, the limitation of materials leads to the single function of optically controlled terahertz devices, yet we have recently conducted some exploratory works on multi-functional and multi-dimensional all-optical terahertz metasurfaces^43–45^. Nevertheless, as opposed to electronic-coding terahertz modulation which is more concerned, optical coding processes have received comparatively little research. Though some efforts excite high-resistance silicon wafers for terahertz imaging using spatial light modulators as encoding masks^46–50^, they mostly focus on single-channel modulation and suffer from low coding speed. Further research is highly required on integrated and miniaturized light-coded metadevices toward programmed ultrafast terahertz switching.

In this work, we have prepared an optically controlled terahertz 2-bit encoding device by combing photonic crystals, i.e., arranged Distributed Bragg Reflector (DBR) microstrips, with terahertz metal metasurface. In terahertz metasurfaces, the multimode coupling effect is intended to provide extreme sensitivity, multi-channel resonances, and enhanced modulation efficiency. Intrinsic epitaxial silicon islands are embedded in split resonant resonators (SRRs) of metal metasurface to control its non-radiative loss for ultrafast modulation behavior. The DBR microstrips that block light at 400 nm and 800 nm tightly integrate with the terahertz metasurface structure and are positioned on the metal metasurface units, resulting in different couple mode dissipations controlled by pump-color excitation. We experimentally tested the 2-bit optical terahertz programming behavior on this integrated metadevice. Mutual verification was conducted through experiments and numerical simulations. In addition, the time-domain coupled mode theory (TCMT) has been proposed to explain the physical mechanism, revealing that the free-standing non-radiative losses of the coupling modes are the foundation of dual channel coding. This optical-programming terahertz-switching method, which has yet to be proposed, is experimentally demonstrated in our metadevice that combines optical metasurface and terahertz metasurface. Our work provides a new paradigm for optically controlled terahertz encoding.

## Results

### Design of color-coded metadevice

**Fig. 1:**
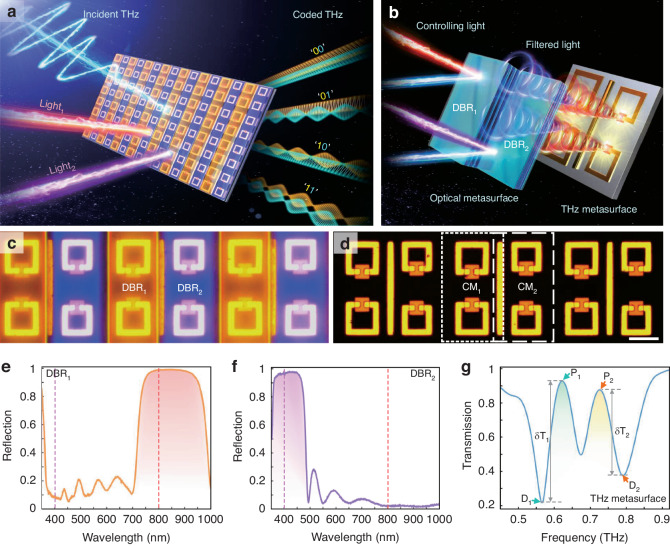
Illustration of the pump color-controlled metadevice for coded terahertz switching. **a** Transmitted terahertz waves can be separately manipulated using two pumped lasers, each carrying different colors. The metadevice is a hybrid with arranged DBRs and a terahertz metasurface. **b** Schematic of a resonating system within one-unit cell supporting multiple coupled terahertz meta-atoms and two precisely aligned DBR mirrors. **c**, **d** Reflective optical micrographs of the fabricated metadevice with and without hybridizing visible metasurface, respectively. Scale bar: 25 μm. The golden section represents the gold metal metasurface, which has detailed geometrical properties as specified in the Supporting Information. The silicon island measures 15 × 15 μm^2^ and is represented by an orange square patch. **e**, **f** Reflectivity spectra for two DBRs corresponding to an area with CM_1_ and for a location with CM_2_. **g** Terahertz transmission spectrum of the color-coded metadevice

Multidimensional electromagnetic responses exist in open multimode systems and the challenge of their independent controlling resides in actively tailoring terahertz waves, that is, simple coupling modes, that maximize their functionality. A sketch of a color-coded metadevice is shown in Fig. 1. Our system consists of a bilayer structure made of coupling terahertz resonators with detuned modes and Distributed Bragg Reflector (DBR) microstrips with two-color spectral filtering. Of interest here is the combination between optical metasurface with dielectric photonic crystal and active terahertz metasurface, which can implement optical encoding terahertz modulation, as shown in Fig. 1b. For the terahertz metasurface, it is vital to realize various resonant features at subwavelength scale, whose lineshapes should be sensitive to the external perturbation. Here, the double electromagnetically induced transparency (EIT) effect with a bright dipole moment coupled with four dark inductive-capacitive resonances is selected to meet these prerequisites. The mode detuning is implemented by breaking the mirror symmetry of a typical EIT meta-system using SRRs of distinct sizes (Fig. 1d), leading to three hybrid modes. Its radiative lineshape is experimentally measured in Fig. 1g. When a 200-nm-thick semiconductor epitaxial layer (silicon, bandgap ~1.1 eV) is incorporated in the SRRs, the terahertz responses can be actively controlled by an external optical excitation. We choose Si as the active material in this case instead of other materials like TMDCs^51,52^, amorphous germanium^40^, perovskite^31^, etc., which are used for all-optical controlled terahertz modulation, for two reasons: (i) The periodic active islands incorporated in SRRs can be fabricated with mature techniques. (ii) More photogenerated carriers of Si can be produced with the same pump fluence, leading to a higher modulation efficiency compared to other semiconductor film materials. For color-coded metadevice, the DBR microstrips with quasi-infinite in the x-y plane and periodic out of the plane are introduced to filter out different pump colors. Here, two DBR microstrips that consist of multiple iterations of Nb_2_O_5_ and SiO_2_ with optimized optical thickness (Table S1 and S2 in the Supporting Information) are built to cover SRRs on both sides of the cut-wire compactly (Fig. 1c). As we can see from the Fig. 1e and Fig. 1f, in experiment the DBR_1_ reflects pump light of 800 nm and transmits that of 400 nm, while the DBR_2_ is exactly the opposite. Upon this, the non-radiative damping rate of each type of coupling metaatom (CM_1_ and CM_2_) can be controlled independently by applying different pumping colors to achieve the encoding of terahertz amplitudes. Our fantastic innovation is the combination of terahertz metasurface with arranged optical-band DBRs. Without DBRs, color coding is not possible, and both terahertz channels will be modulated concurrently with whichever pumping wavelengths. The specific size parameters of the metal metasurface structure are described in detail in the Supporting Information (Fig. S1), and the metadevice processing process can be found in the Methods section.

### Performance of pumping color-coded functionality

**Fig. 2:**
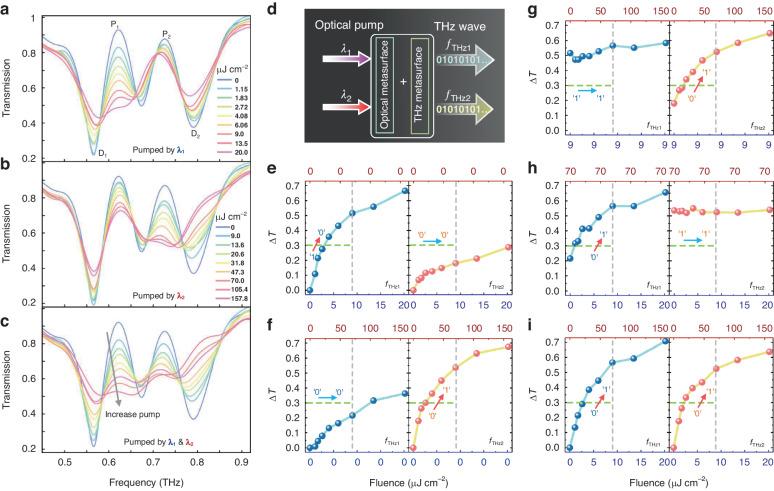
Experimental results of 2-bit color metadevice to demonstrate its ability to control resonance modes by different pumping schemes coded by the wavelength. **a**–**c** THz transmission spectra of the metasurface array under various pump fluences with different coding configurations. **d** A flow diagram for realizing a programmable metasurface controlled by the optical pumping color. **e**–**i** Measured resonance transmission variations of CM_1_ and CM_2_ as a function of the pumping wavelength and fluence, respectively. The Green dashed line represents a transition threshold of two resonance states for different pumping conditions

A multimode system based on the proposed EIT metasurface provides channels for multidimensional information manipulation. To demonstrate the encoding behavior, experimentally extracted magnitude changes at two channels with different excitation wavelengths are described in Fig. 2. Three basic modulation methods can be easily implemented: (i) When the metadevice pumped by ${\lambda }_{1}$ (400 nm), the non-radiative damping of CM_1_ increases and then the EIT resonance of the left channel is suppressed (Fig. 2a). (ii) When the metadevice pumped by ${\lambda }_{2}$ (800 nm), the loss of CM_2_ increases and the right channel is quenched (Fig. 2b). (iii) When the metadevice is simultaneously pumped by ${\lambda }_{1}$ and ${\lambda }_{2}$, both EIT resonances disappear (Fig. 2c). The experimental results found that two channels of EIT resonances can be independently tuned, suggesting a 2-bit pumping color-coded functionality. Note that the frequency of the resonance peak shifts during the modulation process due to the variation in charge accumulation on SRR capacitance-inductance resonance^53^, as well as the near-field effect between coupling metaatoms. Turning now to discuss the encoding process in detail. To distinguish the coding state, we define the state without optical excitation as “0”, which means no terahertz EIT modulation. And the fully modulated EIT with excitation is set as state “1”. When only excited by ${\lambda }_{1}$, the first EIT resonance amplitude defined by peak 1 (P_1_) and dip 1 (D_1_) was effectively tuneable with various pump fluences as shown in Fig. 2e. Similarly, the second EIT resonance amplitude defined by peak 2 (P_2_) and dip 2 (D_2_) was eliminated for ${\lambda }_{2}$ excitation (Fig. 2f). It is evident that a greater pump fluence at 800 nm than at 400 nm is required to achieve maximum modulation. Namely, the pump efficiency of the two channels is different. The reason is that the absorption of 200-nm-thick Si film at different wavelengths varies, resulting in greater photoconductivity in silicon pumped by light at 400 nm than at 800 nm. Simultaneous excitation of two pump wavelengths will result in complete suppression of both EIT resonances (Fig. 2i). We can also alternatively keep one pumping wavelength in the excitation state while changing the pump fluences of the other pumping beam to perform encoding switching in different states (Fig. 2g, h). It should be noted that the crosstalk between the two encodings, indicating that ${\lambda }_{1}$ excitation can influence the second EIT resonance and ${\lambda }_{2}$ excitation can influence the first, which is most likely attributed to the residual impacts of DBR filtering and the coupling of near-field modes. On the one hand, the arranged DBRs are the primary cause of the problem. The grown DBRs cannot achieve a complete reflection of the corresponding wavelength (Fig. 1e, f), and the arranged DBRs also have a diffraction effect (Fig. S5) – a portion of the incident light is dispersed to other channels. On the other hand, the near-field coupling is partially responsible for the crosstalk, since the indirect interaction of two dark modes via a bright mode inside a subwavelength unit cell. The presence of crosstalk will result in the inability to independently encode each channel, which is certainly not what we want. Nevertheless, we can choose a suitable pump fluence (the gray dashed vertical lines) as the excitation to avoid crosstalk, meanwhile setting a threshold (the green dashed horizontal lines) for switching between “0” and “1” states. A 2-bit terahertz encoded modulation is possible when two separate light excitation channels are used, each with a different non-radiation loss of couple mode for independent control.

### Ultrafast terahertz wave switching at 2-bit coding states

**Fig. 3:**
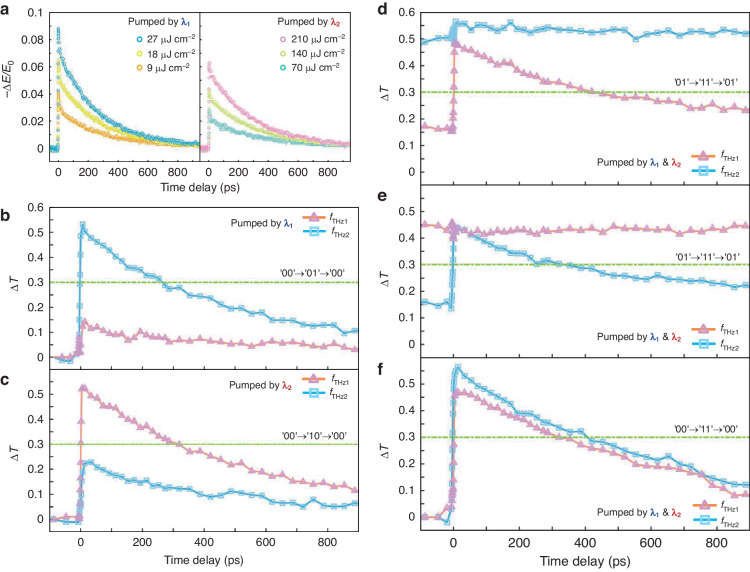
Pumping color-coded ultrafast switching of THz bifunctionality. **a** Differential terahertz transmission revealed the fluence-dependent transient dynamics under two pump laser wavelengths. **b**–**f** Experimental demonstration of ultrafast THz switching of CM_1_ and CM_2_ resonances that are coded through two pumping wavelengths, respectively

We demonstrated the ultrafast dynamic encoding modulation process extracted from time-resolve terahertz spectra with the exploitation of the nanosecond relaxation time of Si islands through femtosecond pulse pumping. The beginning point of light excitation is set at a pump-probe delay time of 0 ps when the photo-generation carrier of Si islands is stimulated. Subsequently, the excited carriers quickly restored to their initial state, along with an ultrafast modulation process of the color-coded metadevice. The transient dynamics of differential terahertz transmissions varying of Si film with the pump fluences under two pump laser wavelengths are shown in Fig. 3a, indicating that higher pump power can achieve greater modulation depth. However, this does not imply that the encoding modulation effect is improved by increasing the magnitude of pump fluence. We need to ensure that there is a clear distinction between “0” and “1”, while simultaneously eliminating the crosstalk precisely caused by the pump interference as mentioned before. Therefore, the selection of an adequate pump fluence and switching threshold is taken into account. The threshold is chosen based on crosstalk in experimental testing to ensure that there is no interference between the two channels during the encoding process. Specifically, we must make sure that the green dashed lines in Fig. 2e–h do not cross the orange and blue curves simultaneously. In the experiment, we select pump fluence of 9 $\mu J\,{{cm}}^{-2}$ at ${\lambda }_{1}$ and that of 70 $\mu J\,{{cm}}^{-2}$ at ${\lambda }_{2}$ as an excitation strategy for color encoding. The switching threshold between “0” and “1” states is set as 0.3 of differential EIT resonance amplitude (ΔT). Specifically, ΔT is less than 0.3 in the “0” state, while it is more than 0.3 in the “1” state. In this regard, we can claim that all-optical encoding for independently controlling the loss of CMs in binary channels with two stimulating wavelengths has been successfully achieved. The ultrafast modulation processes of differential EIT resonance amplitude within 1000 ps are measured by utilizing optical-pump terahertz-probe (OPTP) technology. By moving the relative time between the pump lights and the terahertz pulse arriving at the surface of the metadevice, the frequency-domain spectral as a function of time delay can be obtained. We show the ultrafast dynamic evolution of multiple encoding switches for various pump modulation approaches as illustrated by Fig. 3b–f. This behavior is based on the ultrafast terahertz switching of CM_1_ and CM_2_ resonances that are coded through two pumping wavelengths, respectively.

**Fig. 4:**
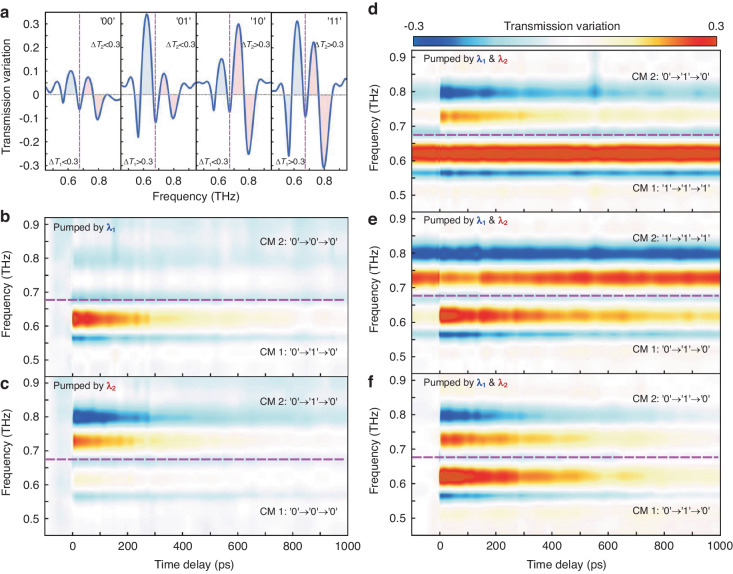
Ultrafast THz response and spectral variation dynamics under different pumping configurations. **a** Measured THz transmission spectral variation at various coded states. **b**–**f** Pumping color driving THz spectral variation evolutions over the entire ultrafast on-off-on photoswitching cycle

To better highlight the modulation changes brought out by the optical pump excitation, the transmission variation is defined as ${T}_{v}\left(\omega \right)={T}_{{without\; pump}}\left(\omega \right)-{T}_{{with\; pump}}\left(\omega \right)$. Figure 4a depicts the EIT resonance transmission variations for the four coding states (“00”, “01”, “10”, “11”) in accordance with the standard of switching threshold. For the initial state of the metadevice at “00”, the terahertz transmission spectrum, without pumping excitation, exhibits a dual-channel EIT resonance curve. The encoding process is 00-01/10-00 when each color (*λ*_1_ *or λ*_2_) is excited independently (Fig. 4b, c), while the red and blue parts mapped in Fig. 4 indicate the variations modulated by the pump, showing an ultrafast on-off-on photo-switching cycle within 1000 ps. When two color lights are excited simultaneously, the ultrafast encoding modulation is 00-11-00 (Fig. 4f). Also, alternatively activating one channel while modulating the other allows us to achieve an encoding process of 01/10-11-01/10 (Fig. 4d, e). Subsequently, we validated the transient dynamics of different coded states through the findings of numerical simulation. The variation of Si conductivity with time delay is first extracted from the experimentally measured transient differential terahertz transmission and fitted by bi-exponential functions (Fig. S2, Supporting Information), and then it is substituted into the finite element method simulation with COMSOL Multiphysics for numerical calculations. The entire ultrafast simulation can be regarded at different points in time as a quasi-steady state process since the relaxation time of silicon is far longer than a terahertz pulse’s duration. The simulated terahertz transmission spectral variation at various pump-color-coded states described in Fig. S3 (Supporting Information) agrees well with the experimental results. Here we have implemented a light-control 2-bit ultrafast terahertz encoding operation, which has not been done before. In addition, the near-field distribution profiles of terahertz metasurface for different coded states are simulated in Fig. S4, Supporting Information. The discoveries that the localized electric field at the gap of the SRRs descends when the conductivity of the Si islands increases for the two couple modes provide insight into the microscopic mechanism of encoding. Besides, the changes in the electric field strength of one SRR mode affect that of the other SRR, hence influencing the resonance intensity of the two channels simultaneously. The distribution of the pump light field in DBRs also shows that optical beams with 800 nm and 400 nm excite difference channels, respectively (Fig. S5, Supporting Information). This reveals that the recombination of arranged DBRs and dual-EIT terahertz metasurface is the source of the 2-bit terahertz encoding’s functionality, delivering a unique method for optically encoding terahertz information via terahertz metasurface in conjunction with optical metasurface.

### Theoretical interpretation of 2-bit coding

The performance of 2-bit terahertz encoding originates from the modulation of non-radiative loss of two dark modes on SRRs, we can use the time-domain coupled mode theory (TCMT) to theoretically explain and fit the modulation of the spectral curves, which can be expressed as:

1 $$\frac{1}{2\pi }\frac{d{\boldsymbol{a}}}{{dt}}=\left(j\Omega -\Gamma \right){\boldsymbol{a}}+{D}^{T}{{\boldsymbol{s}}}_{+}$$

2 $${{\boldsymbol{s}}}_{-}=C{{\boldsymbol{s}}}_{+}+D{\boldsymbol{a}}$$

where ${\boldsymbol{a}}{\boldsymbol{=}}{\left({a}_{p},{a}_{m},{a}_{n}\right)}^{T}$ is the vector form of complex amplitudes of three resonance modes, ${{\boldsymbol{s}}}_{{\boldsymbol{+}}}{\boldsymbol{=}}{{\boldsymbol{(}}{s}_{+}^{1}{\boldsymbol{,}}{s}_{+}^{2}{\boldsymbol{)}}}^{T}$ and ${{\boldsymbol{s}}}_{{\boldsymbol{-}}}{\boldsymbol{=}}{({s}_{-}^{1},{s}_{-}^{2})}^{T}$ are the input and output amplitudes of each port, Ω is the matrix that describes the resonance frequencies and the near-field coupling coefficients, *D* is the coupling coefficients between resonances and input and output ports, Г is the matrix of the dissipation rates and the indirect coupling coefficients. The terahertz metasurface can be modeled as a three-mode two-port system, we further define the matrices as^54^:

3 $$\Omega =\left(\begin{array}{ccc}{f}_{p} & {{\kappa }}_{{pm}} & {{\kappa }}_{{pn}}\\ {{\kappa }}_{{pm}} & {f}_{m} & {{\kappa }}_{{mn}}\\ {{\kappa }}_{{pn}} & {{\kappa }}_{{mn}} & {f}_{n}\end{array}\right)\Gamma =\left(\begin{array}{ccc}{\gamma }_{p+}{\gamma^\prime_p} & 0 & 0\\ 0 & {\gamma }_{m+}{\gamma^\prime_m} & {{\rm{X}}}_{{mn}}\\ 0 & {{\rm{X}}}_{{mn}} & {\gamma }_{n+}{\gamma^\prime_n}\end{array}\right)$$

4 $$C=\left(\begin{array}{cc}{r}_{{sub}} & {t}_{{sub}}\\ {t}_{{sub}} & {-r}_{{sub}}\end{array}\right)\,D=\left(\begin{array}{ccc}{d}_{1p} & {d}_{1m} & {d}_{1n}\\ {d}_{2p} & {d}_{2m} & {d}_{2n}\end{array}\right)$$

where $\gamma$ and ${\gamma }^{{\prime} }$ is the radiative and non-radiative loss, ${r}_{{sub}}=\left(1-{n}_{{sub}}\right)/\left(1+{n}_{{sub}}\right)$ and ${t}_{{sub}}=2\sqrt{{n}_{{sub}}}/\left(1+{n}_{{sub}}\right)$ denote the reflection and transmission of the bare substrate, respectively. Then we can obtain the expression of the transmission curve $t={s}_{-}^{2}/{s}_{+}^{1}$ by calculating the coupled mode equation. ${X}_{{mn}}$ is the far-field indirect coupling of modes ${a}_{m}$ and ${a}_{n}$. Considering that the terahertz polarization direction follows the metal cut-wire, ${a}_{m}$ and ${a}_{n}$ are dark modes with no external coupling, therefore, ${X}_{{mn}}=0$, ${d}_{1m}={d}_{1n}={d}_{2m}={d}_{2n}=0$. Ignoring the direct near-field coupling between two dark modes (${\kappa }_{{mn}}=0$), then the complex transmission coefficient can be obtained,

5 $$t={t}_{{sub}}-\frac{2\sqrt{{n}_{{sub}}}{{\rm{\gamma }}}_{p}}{1+{n}_{{sub}}}\frac{{W}_{m}{W}_{n}+{{\rm{\kappa }}}_{{mn}}}{{W}_{p}{W}_{m}{W}_{n}+{W}_{m}{{\rm{\kappa }}}_{{pn}}^{2}+{W}_{n}{{\rm{\kappa }}}_{{pm}}^{2}}$$

where $${W}_{j}=-i(f-{(f}_{j}-i({\gamma }_{j}+{\gamma }_{j}^{{\prime} })))$$ $$\left(j=p,\,m,\,n\right)$$.

**Fig. 5:**
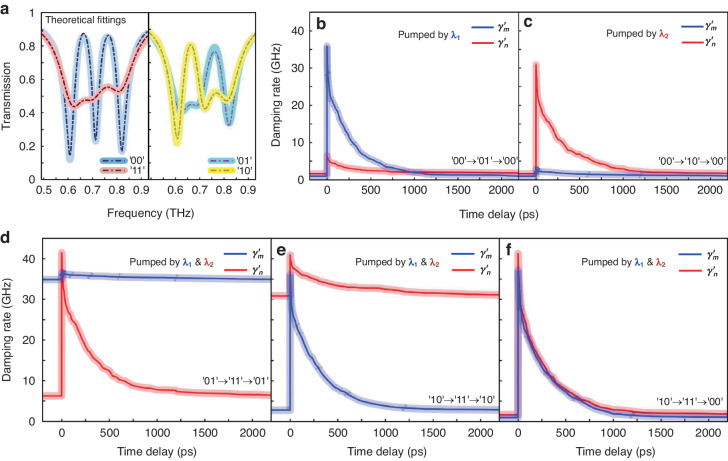
Theoretical interpretation of temporal loss dynamics and physical elaboration of switching coded states. **a** TMCT fitted transmission spectra for different coded states. **b**–**f** Nonradiative damping rates of CM_1_ and CM_2_ that are tightly associated with pumping conditions as well as transient time delay

Accordingly, we fitted the four encoding states examined in the experiment using the TCMT model (Fig. 5a), where the transparent solid lines indicate the fitting findings and the dotted lines represent the experimental data. Using numerical simulation, we first estimate the values of the coupling mode parameters and then make adjustments in line with the experimental results. We approach the transmission curves determined by the coupling mode theory to the experimental results using a genetic algorithm. Subsequently, the coupling mode parameters can be generated that are extremely near to the experimental curves. The detailed parameters for the four encoding states are listed in Table S3 in the Supporting Information. The TCMT model can accurately reproduce experimental results because it considers the interactions between various modes and the surrounding environment. Figure 5b–f illustrates how the extracted nonradiative damping rates of two dark modes (${\gamma }_{m}^{{\prime} }$, ${\gamma }_{n}^{{\prime} }$) change during time delay under various pump excitations, which correspond to the ultrafast encoding switching of the simulated calculations and experimental observations, respectively. The transient behavior of other parameters can also be found in Supporting Information (Fig. S6). The theoretical interpretation explains that transient non-radiative losses of CM_1_ and CM_2_ are the physical causes of the control of 2-bit ultrafast encoding. It is helpful to provide direction for the design of controllable lineshapes and corresponding function implementation.

## Discussion

In summary, we proposed a novel method capable of all-optically controllable 2-bit terahertz encoding by combining photonic crystals and terahertz metasurface. The three-mode coupling supported by the terahertz metasurface allows for a dual-channel resonance spectrum and provides active loss-sensitive modulation. Additionally, the strategically placed DBRs create a particular spatial light distribution that filters respectively out the 400 nm and 800 nm pump wavelengths for color-pump terahertz switching. Through experimental testing, we were able to demonstrate the ultrafast behavior of encoding switching at the nanosecond scale, which excellently aligns with the intended effects of simulations. Moreover, the TCMT model provides a proper explanation for the encoding principle, which originates from the tailoring of non-radiative losses of the dissipative couple modes. Our strategy provides a new development way for optical coding terahertz modulation and further inspires the exploration of optical programming terahertz devices based on metasurfaces. This coding approach can be extended to multi-bit information processes and may be applied in the field of optical-controlled terahertz imaging.

## Materials and methods

### Sample fabrication

In the schematic of Fig. 1b, the terahertz metasurface is on a 1-mm-thick sapphire substrate and embedded with 200-nm-thick silicon islands. The second layer is on top of the z-cut quartz substrate and precisely adhered to the terahertz metasurface, making its DBRs filter the corresponding pumping wavelengths. The terahertz resonators are fabricated using two-step photolithography and metal lift-off on a commercially available silicon-on-sapphire (SoS) substrate. Positive photoresist spin-coated on SoS is first patterned with silicon islands via a photomask, followed by lithography and development. The unprotected silicon is subsequently etched by reactive ion etching. The metallic array patterns precisely aligned with silicon islands are defined in the photoresists, followed by 200-nm-thick gold deposition and a lift-off process. The wavelength filtering sample consists of two kinds of DBRs, each with 8 periods of SiO_2_ & Nb_2_O_5_ in the z-direction, that are separated by a 5-μm spacer in the y-direction. The DBRs are fabricated by magnetron sputtering coating equipment on a quartz substrate using an Nb_2_O_5_ composite target and Si composite target in O_2_ gas with a substrate temperature of 200 °C. Unlike conventional DBR mirrors, our proposed DBRs are fabricated with an additional procedural photolithography technique to define the patterns of DBR_1_ and DBR_2_. For detailed structure parameters please see section 1 in the Supporting Information. An intuitive flowchart of sample fabrication processing is shown in Fig. S7.

### Optical pump–terahertz probe spectroscopy

We use a home-built optical pump terahertz-probe spectroscopy setup to perform transmission measurements so that time-resolved terahertz spectra can be obtained. Femtosecond pulses generated from a Ti: Sapphire amplifier laser have a central wavelength of 800 nm, repetition rate of 1 kHz, and pulse width of ~100 fs. Linearly polarized terahertz waves are produced from a ZnTe crystal when illuminated by a femtosecond laser pulse train. The horizontal electric field of the transmitted terahertz wave is detected using a typical electro-optic sampling method in the time domain by a ZnTe crystal. To realize color-selective multifunctionality, the pumping beam is divided into two parts, one directly illuminating the metadevice and another one passing through a BBO crystal to generate a 400 nm femtosecond laser. The diameter of the pumping beam is ~5 mm which covers the terahertz spot uniformly on the sample. The whole setup is purged with dry gas to prevent the fingerprint water vapor absorption of terahertz waves. Far-field spectral amplitudes are derived from the measured data via numerical Fourier transformation and normalized with the reference signal of a pure substrate.

### Electromagnetic simulation

Numerical simulations are executed using the finite element method (COMSOL Multiphysics^®^) to calculate the transmitted coefficients of the periodic terahertz resonators. The periodic feature of meta-atoms is modeled using a unit cell with periodic boundary conditions in x- and y-directions, representing infinity in the xy plane. Two perfectly matched layers are selected in the z-direction to absorb the remaining energy after the field monitor. As for different coded states, the photoconductivity of silicon islands in CM_1_ and CM_2_ are independently controlled. In transient dynamic simulations, the time-dependent conductivity of silicon islands is substituted into the solver based on the formula $\Delta \sigma \left(t\right)={\varepsilon }_{0}c/d\left({n}_{a}+{n}_{s}\right)\left[-\Delta E\left(t\right)/{E}_{0}\right]$. Here, $-\Delta E\left(t\right)/{E}_{0}$ is the fitted terahertz transmission amplitude variation by pumping a 200-nm-thick silicon film, ${n}_{a}$ is the refractive index of air and ${n}_{s}$ is the refractive index of the sapphire substrate. The *c* is the speed of light in a vacuum, *d* is the thickness of silicon film, and ${\varepsilon }_{0}$ is the vacuum permittivity constant. The conductivity of the gold is described using a Drude model with a plasma frequency *ω*_*p*_*=*1.367 × 10^16^ rad s^−1^ and a collision frequency *ω*_c_ = 6.478 × 10^13^ rad s^−1^. For the substrates, z-cut quartz and sapphire are modeled as a lossless dielectric with a permittivity of 3.9 and 9.6, respectively. The silicon islands without photoexcitation are set with a permittivity of 11.7.

### Supplementary information

Revision-Supporting Information
