# Supplementary material for: Color coded metadevices toward programmed terahertz switching
Source: Light Sci Appl. 2024 Jun 25;13:142. doi: 10.1038/s41377-024-01495-1 (PMC11196690; doi:10.1038/s41377-024-01495-1)
Supplement: Supplementary file 1 — Revision-Supporting Information [file 41377_2024_1495_MOESM1_ESM.pdf]

Supporting Information for

Color Coded Metadevices toward Programmed Terahertz Switching

Weibao He[1,2,3], *Xiang'ai Cheng*[1,2,3], *Siyang Hu*[1], *Ziheng Ren*[1], *Zhongyi Yu*[1], *Shun Wan*[1], *Yuze Hu*[*,4], *Tian Jiang*[*,4]

[1] College of Advanced Interdisciplinary Studies, National University of Defense Technology, Changsha 410073, China
[2]Nanhu Laser Laboratory, National University of Defense Technology, Changsha 410073, China
[3]Hunan Provincial Key Laboratory of High Energy Laser Technology, National University of Defense Technology, Changsha 410073, China
[4]Institute for Quantum Science and Technology, College of Science, National University of Defense Technology, Changsha 410073, China

Correspondence: Professor Yuze Hu, E-mail: hyz_yj@sina.com
Correspondence: Professor Tian Jiang, E-mail: tjiang@nudt.edu.cn

1. Detailed geometrical parameters for the rainbow-controlled metadevice

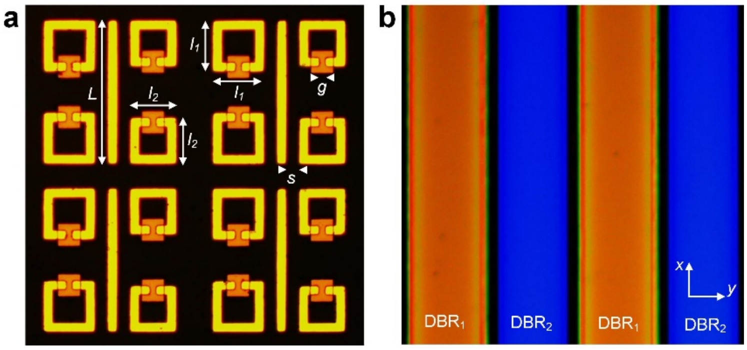

Figure S1 Optical micrographs of the (a) THz metasurface and (b) periodically distributed DBRs. The key geometrical parameters are $L = 85$ μm , $l_1 = 30$ μm , $l_2 = 27$ μm , $s = 7$ μm , $g = 5$ μm . The metal wire width is 5 μm. The periodicities of THz metasurface are $P_x = 100$ μm , $P_y = 100$ μm , respectively.

The proposed metadevice consists of two-layered microstructures. The THz metasurface is hybridized with a 200-nm-thick gold pattern and 200-nm-thick silicon islands. Detailed geometric parameters are given in Figure S1. As for the DBRs, it would become more complicated. In the xy-plane, DBR_1 and DBR_2 are periodically distributed along the y-direction with a periodicity of 50 μm and a spacer of 5 μm. In *z*-direction, both DBRs are fabricated using the deposition technique with multiple iterations of SiO_2 and Nb_2O_5. The corresponding processing parameters are provided in Table S1 and Table S2, respectively.

Table S1. The experimental parameters of DBR_1

Layer Num.	Material	Refractive Index	Optical Thickness (FWOT)	Physical Thickness (nm)
Substrate	Quartz	1.50908	----	----
1	Nb_2O_5	2.27034	0.25	88.09
2	SiO_2	1.47132	0.25	135.93
3	Nb_2O_5	2.27034	0.25	88.09
4	SiO_2	1.47132	0.25	135.93
5	Nb_2O_5	2.27034	0.25	88.09
6	SiO_2	1 47132	0.25	135.93
7	Nb_2O_5	2.27034	0.25	88.09
8	SiO_2	1.47132	0.25	135.93
9	Nb_2O_5	2.27034	0.25	88.09
10	SiO_2	1.47132	0.25	135.93
11	Nb_2O_5	2.27034	0.25	88.09
12	SiO_2	1.47132	0.25	135.93
13	Nb_2O_5	2.27034	0.25	89.09
14	SiO_2	1.47132	0.25	135.93
15	Nb_2O_5	2.27034	0.25	88.09
16	SiO_2	1.47132	0.25	135.93

Table S2. The experimental parameters of DBR_2

Layer Num.	Material	Refractive Index	Optical Thickness (FWOT)	Physical Thickness (nm)
Substrate	Quartz	1.50908	----	----
1	Nb_2O_5	2.27034	0.29340674	69.55
2	SiO_2	1.47132	0.25341835	68.06
3	Nb_2O_5	2.27034	0.24606834	39.19
4	SiO_2	1.47132	0.27321025	73.37
5	Nb_2O_5	2.27034	0.25415032	40.47
6	SiO_2	1 47132	0.28491142	71.14
7	Nb_2O_5	2.27034	0.27497685	43.79
8	SiO_2	1.47132	0.30215537	81.14
9	Nb_2O_5	2.27034	0.25614756	40.79
10	SiO_2	1.47132	0 22200308	59.62
11	Nb_2O_5	2.27034	0 27666799	44.06
12	SiO_2	1.47132	0.28440869	76.38
13	Nb_2O_5	2.27034	0.26963412	42.95
14	SiO_2	1.47132	0.19420114	52.15
15	Nb_2O_5	2.27034	0.25666387	40.87
16	SiO_2	1.47132	0.27409753	73.61

2. Numerical calculations for the transient dynamics of different coded states

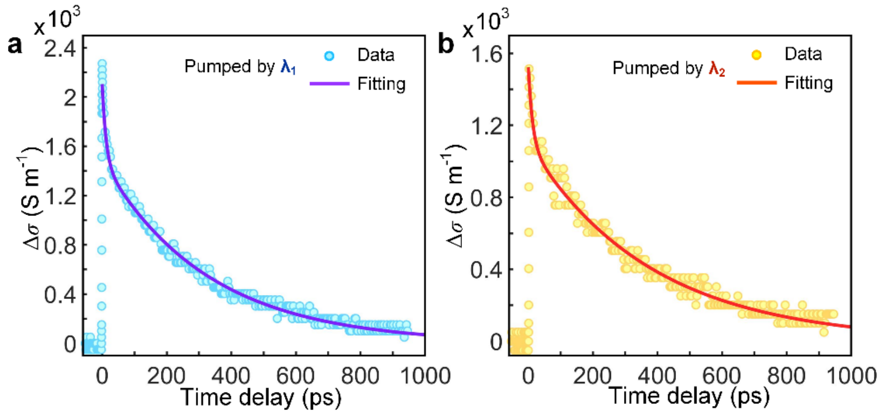

Figure S2 Transient photoconductivity of the photoexcited 200-nm-thick silicon film under two pumping wavelengths of (a) 400 nm and (b) 800 nm. The solid curves are fitted by biexponential decay functions.

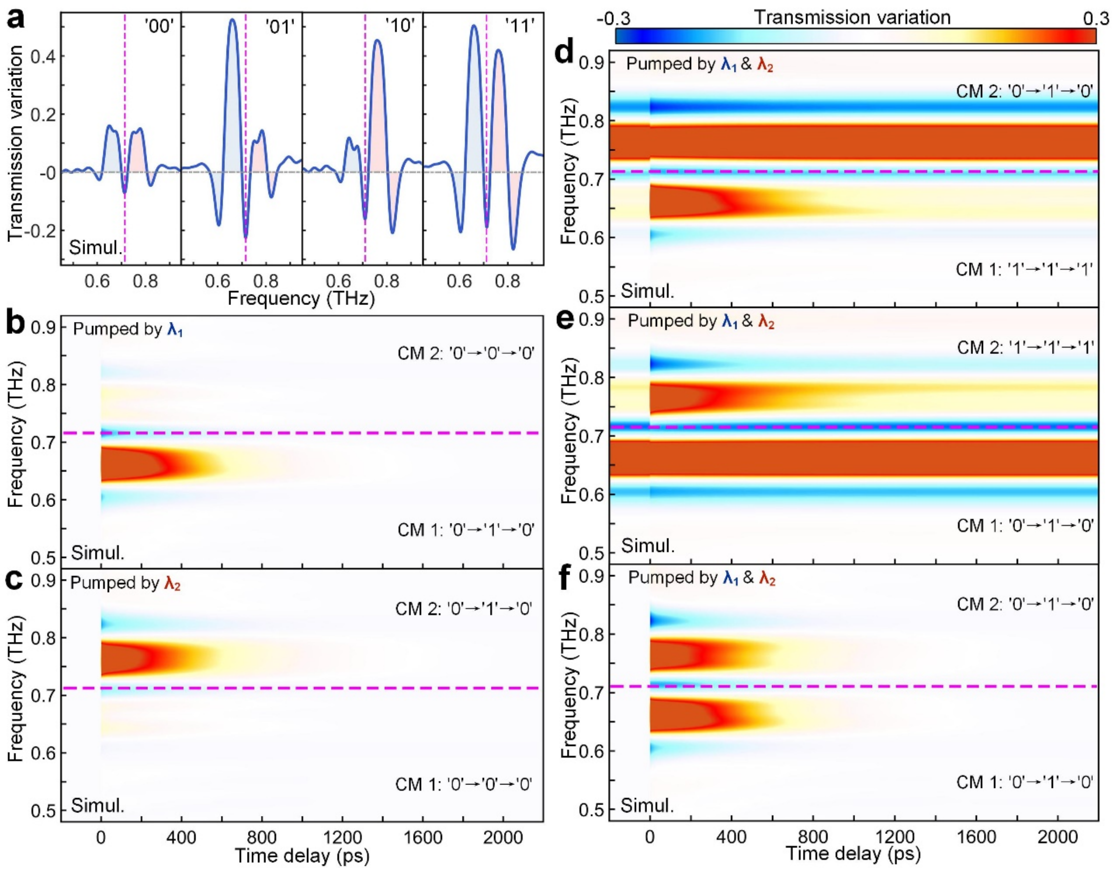

Figure S3 Ultrafast THz response and spectral variation dynamics under different pumping configurations. (a) Simulated THz transmission spectral variation at various coded states. (b)-(f) Pumping color driving THz spectral variation evolutions over the entire ultrafast on-off-on photoswitching cycle that are obtained by numerical calculations.

3. Near-field distributions of THz metasurface in the *xOy* cross-section for different coded states

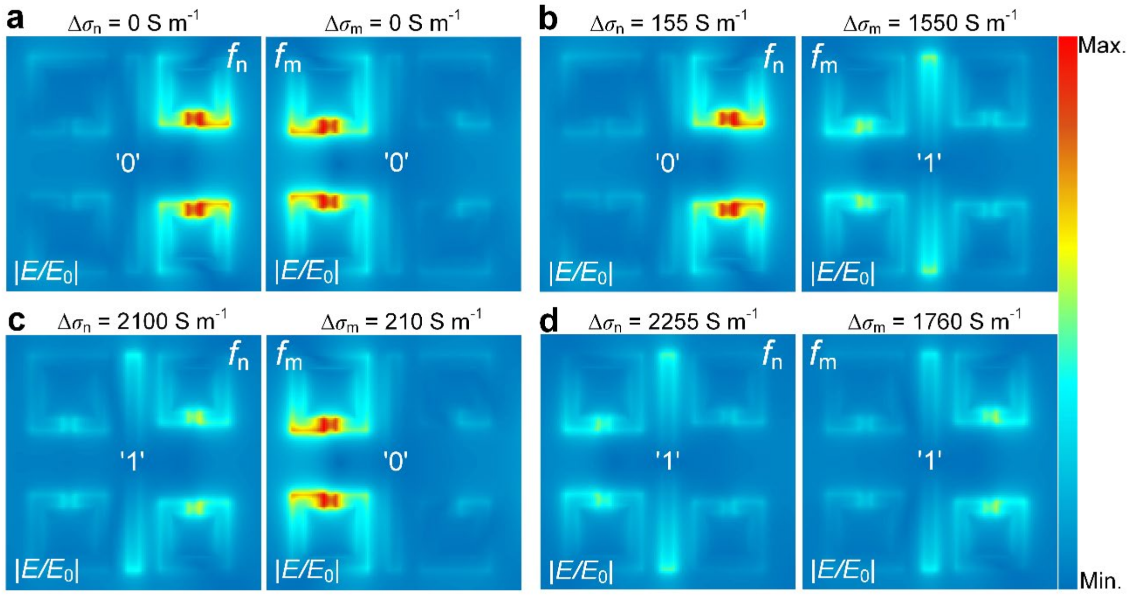

Figure S4 Electric field profiles for the THz metasurface with different excitation states. (a)-(d) Electric amplitude profiles of CM_1 at f_n and CM_2 at f_m for four coded states '00', '01', '10', and '11', respectively.

4. Pumping light distributions in the *yOz* cross-section when filtered by the DBRs

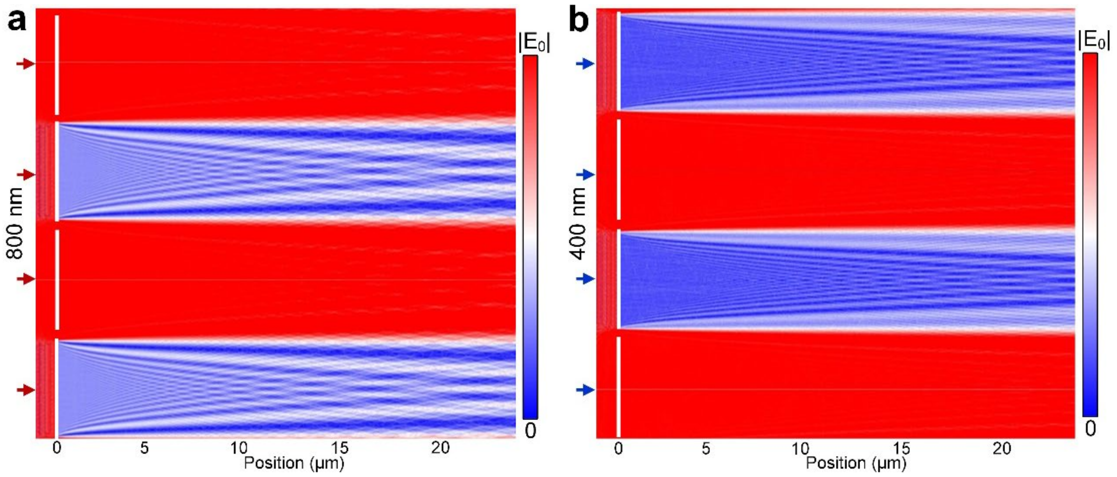

Figure S5 Electric field intensity in the yOz cross-section when the metadevice is pumped by (a) 800 nm and (b) 400 nm optical beams.

5. The parameters of TCMT fitting for different coded states

Table S3. List of mode parameters for four encodings. Unit: Hz. $\gamma_p' = 0.04$ THz.

code	00	01	10	11
f_p	710795511690.279	708146955259.442	709397297440.142	707459606841.654
f_m	662083097728.262	662111356701.780	661293444720.073	658693651875.081
f_n	767261433414.699	767654175728.494	770889000755.940	767602888409.933
γ_p	77487335465.019	82069807600.385	81285355853.905	81877629092.629
γ_m	5583849310.727	25373665271.820	8381227498.776	22211855827.895
γ_n	6358876587.093	11363337238.774	27432320755.401	31569851402.878
γ_m'	1016808403.555	35939270375.142	2814783708.875	37154905968.667
γ_n'	1697856364.172	6555430996.212	30849304148.099	41244929122.749
κ_{pm}	64009151743.115	60424297798.886	62559641825.557	53085683146.370
κ_{pn}	66573476121.820	68340485137.453	67947602201.431	76111432410.798

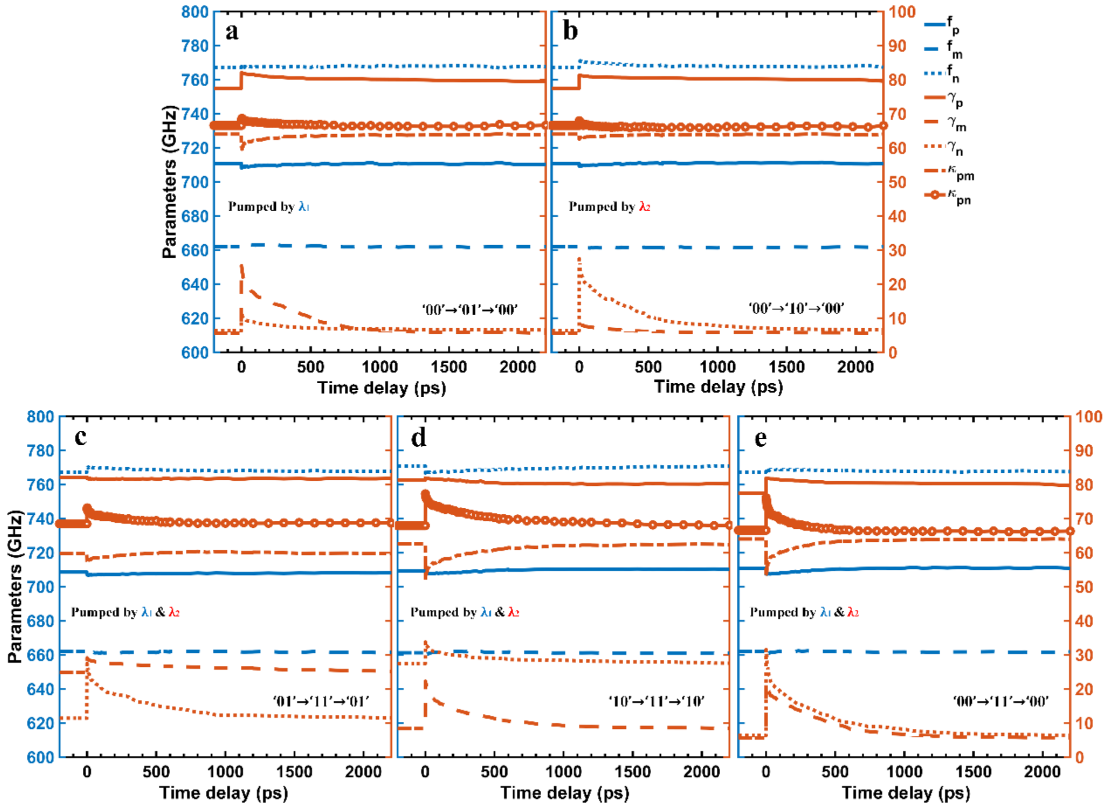

Figure S6. The transient behavior of resonance frequencies: f_p, f_m, f_n, radiative damping rates of resonance modes: γ_p, γ_m, γ_n, and coupling coefficients between bright mode and dark modes: κ_{pm}, κ_{pn}.

6. The fabrication process of THz metasurface and optical DBRs

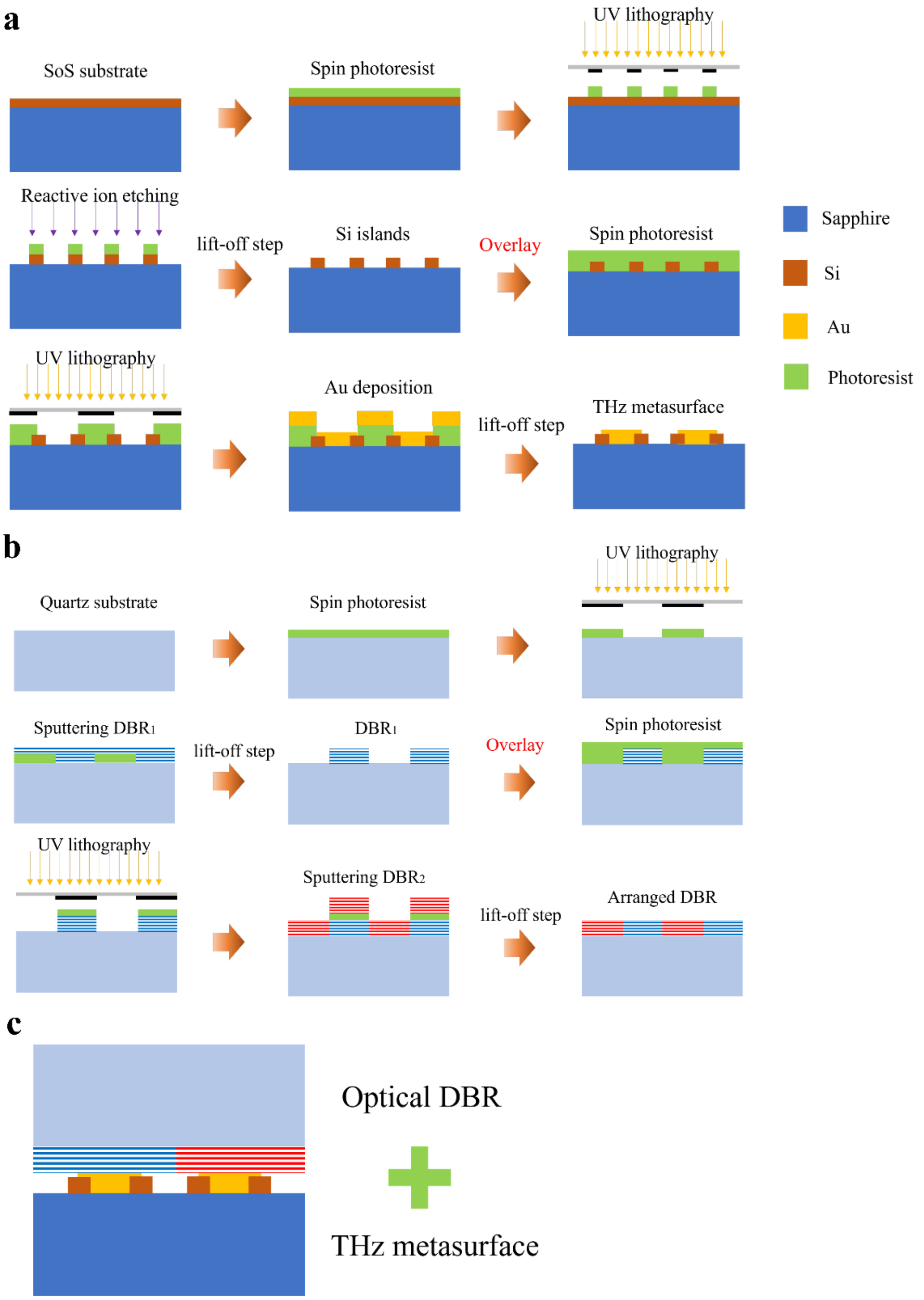

Figure S7. The fabrication process of (a) Si-hybrid terahertz metal metasurface and (b) arranged DBRs. (c) The combination of optical metasurface and active terahertz metasurface. We align two channels under microscope operation in the experiment.
